# Establishment of a two-tiered molecular identification system for *Dracaena* species – the source plants of dragon’s blood

**DOI:** 10.3389/fpls.2026.1851019

**Published:** 2026-07-16

**Authors:** Yuxiu Zhang, Haiying Wu, Xinquan Yang, Yan Zeng, Menglian Feng, Junna Zhang, Yuling Ye, Peiwei Liu, Jianhe Wei

**Affiliations:** 1 Key Laboratory of Resources Conservation and Development of Southern Medicine of Hainan Provincial, Hainan Branch of the Institute of Medicinal Plant Development, Chinese Academy of Medical Sciences & Peking Union Medical College, Haikou, China; 2 State Key Laboratory of Bioactive Substance and Function of Natural Medicines, Institute of Medicinal Plant Development, Chinese Academy of Medical Sciences & Peking Union Medical College, Beijing, China

**Keywords:** DNA barcode, *Dracaena*, dragon’s blood, molecular identification, SSR molecular marker

## Abstract

**Background:**

The unresolved taxonomy of *Dracaena* species, the botanical sources of the prized traditional medicine Dragon’s blood, poses significant challenges to resource conservation, material traceability, and quality control. This study aimed to resolve these taxonomic ambiguities by developing and validating a high-resolution, integrated molecular authentication system. Based on 26 original plant samples from the genus *Dracaena*, along with relevant DNA barcode loci retrieved from NCBI, five previously reported DNA barcodes were evaluated and assessed. Simple sequence repeat (SSR) sequences from the transcriptome of *D. cambodiana* were subsequently screened. Finally, the established DNA barcode and SSR molecular marker systems were applied to identify the botanical origin of Dragon’s blood.

**Results:**

The *trnP-psaJ* and *psbK-psbI* regions were identified as optimal barcodes, effectively distinguishing foreign sources (*D. cinnabari* and *D. draco*) from domestic ones. From transcriptomic data, we screened and validated 16 highly polymorphic SSR primers. While DNA barcodes confirmed broad phylogenetic relationships, SSR markers provided superior resolution, clearly delineating the closely related domestic taxa *D. cambodiana*, *D. cochinchinensis*, and the “*Yanzong*” plant into distinct genetic clusters. A key finding was the consistent genetic distinctiveness of Hainan Island populations, supporting the genetic affinity of the Hainan populations with D. cambodiana, indicating that they fall within the genetic variation range of this species based on the available reference samples. Furthermore, cultivation analysis revealed that *D. cambodiana* is the predominant cultivated species in China; the horticultural variety “Heizhenzhu” was identified as a likely variant of this species, while “Taikongtie” showed ambiguous affinity, possibly involving *D. cochinchinensis* or Guangxi populations.

**Conclusions:**

We established a novel, two-tiered molecular identification system that combines the *trnP-psaJ* barcode for rapid screening with a panel of 16 SSR markers for fine-scale discrimination. This integrated approach successfully clarifies the taxonomy of important Dragon’s blood source plants. It provides a reliable, efficient protocol for authenticating *Dracaena* species and germplasm, offering a critical technical foundation for resource conservation, medicinal material standardization, and targeted breeding programs.

## 1 Introduction

Dragon’s blood has a long history of medicinal use across diverse civilizations, including ancient Greece, Rome, Arabia, and China (Celiński et al., 2022; Liu et al., 2021; Huang et al., 2021). In China, its documented use dates back to more than 1500 years ago. Renowned for its unique efficacy in promoting blood circulation, resolving stasis, promoting tissue regeneration, and healing wounds, it was honored as the “Holy Medicine for Activating Blood” in the *Compendium of Materia Medic*a (Cai and Xu, 1979; Xie, 1989). The red resin exuded from the wounded trunks of *Dracaena cinnabari* Balf.f. and *D.draco* (L.) was the original source of dragon’s blood (Cai and Xu, 1979; Xin et al., 2024; Xie, 1989). In fact, a total of 26 plant species from 4 families have been documented as the botanical sources of Dragon’s blood (Gupta et al., 2008). Dragon’s blood refers to the crimson resinous secretion from plants of the *Dracaena* genus, or saps from species of other distinct genera, including *Pterocarpus*, *Croton*, and *Daemonorops* (Gupta et al., 2008; Liu et al., 2021). Among these, *Dracaena* species are the original and most widely used botanical source, while saps from other genera are considered later substitutes. Although their sources have varied over time, plants of the genus *Dracaena* remain the primary botanical resource for obtaining dragon’s blood (Liu et al., 2021; Celiński et al., 2022).

The genus *Dracaena*, belonging to the family Asparagaceae, comprises approximately 190 species. However, its taxonomic boundaries and nomenclature remain contentious (Maděra et al., 2020; Celiński et al., 2020; Takawira-nyenya et al., 2018). Currently, the raw material for dragon’s blood in China still relies heavily on imports from Southeast Asian countries such as Laos and Vietnam. In local trading markets, all *Dracaena* species that produce red resin are used as source materials for dragon’s blood extraction, often neglecting an accurate botanical identification and leading to significant confusion regarding the commercial sources of dragon’s blood (Zhang et al., 2021).

To address the dependence on imports, Chinese scientists discovered that the red resin produced by the domestically distributed species *D. cochinchinensis* (Lour.) S. C. Chen and *D.cambodiana* Pierre ex Gagnep. possesses a chemical composition and pharmacological activities similar to those of foreign-produced dragon’s blood (Liu et al., 2021; Cai and Xu, 1979). This discovery led to the development of domestic “dragon’s blood” medicinal material (Tianjin medical products administration, 2022; Hainan food and drug administration, 2011; National food and drug administration, 1999). All dragon’s blood-producing *Dracaena* species are perennial trees. During their juvenile stage, their leaf shapes are similar and can change according to the environmental conditions, making them extremely difficult to distinguish using traditional morphological methods in the absence of flowers or fruits (Celiński et al., 2020; Pang et al., 2020). Furthermore, the names used for *D. cochinchinensis, D. cambodiana*, and “*Yanzong*” (a vernacular name referring to an unidentified perennial tree of the genus *Dracaena*, which is used to produce dragon’s blood) distributed within China are often confused (Xin et al., 2024; Zhang et al., 2021, 2019). According to our survey, other ornamental cultivars in China include “Baihezhu”, “Xianglongxueshu”, “Heizhenzhu”, and “Taikongtie”. These factors collectively exacerbate the confusion surrounding the botanical origin of dragon’s blood, which not only constrains the development and utilization of dragon’s blood resource plants but also poses potential risks to clinical medication safety, seriously hindering the healthy development of the dragon’s blood industry. Therefore, standardizing the nomenclature of dragon’s blood source plants and establishing a scientific and efficient rapid identification system are imperative.

Researchers worldwide have attempted to identify *Dracaena* species using various molecular methods, such as DNA barcoding (Zhang et al., 2021; Pang et al., 2020; Wang et al., 2015) or chloroplast genomics (Zhang et al., 2019; Celiński et al., 2022; Ahmad et al., 2022; Xin et al., 2024). Due to the short length of DNA fragments and limited genetic information, the effectiveness of DNA barcodes for identifying closely related species is often unsatisfactory. Conversely, chloroplast genome studies involve higher technical requirements and costs, limiting their practical application. Despite providing more genetic information than single barcodes, chloroplast genomes have limited resolution for closely related *Dracaena* species due to their conserved nature (Zhang et al., 2019; Celiński et al., 2020; Xin et al., 2024). SSR markers offer higher polymorphism, co-dominant inheritance, and better resolution for intraspecific and closely related species discrimination, which is valuable for future breeding programs. This study combines DNA barcoding and SSR molecular marker technologies to identify and authenticate the source plants of dragon’s blood and explore their intrinsic genetic relationships, aiming to provide scientific data for the conservation and development of dragon’s blood resource plants. While direct molecular authentication of Dragon’s blood resin remains challenging due to DNA degradation and complex secondary metabolites, identifying its potential source plants (*Dracaena* species) represents a practical and necessary intermediate step.

## 2 Materials and methods

### 2.1 Materials

The plant materials collected for this study were divided into two groups: The first group consisted of 26 samples, as well as horticultural varieties such as “Baihezhu”(BHZ), “Xianglongxueshu”(XLXS), “Heizhenzhu”(HZZ), and “Taikongtie” (TKT). These samples were primarily used for screening DNA barcode sequences and SSR core loci and for constructing the phylogenetic tree (Samples 1–26 in appendix **Table 1**). The second group comprised cultivated Dracaena trees collected from Hainan, Guangdong, and Yunnan. All samples were formally identified by Peiwei Liu and Xinquan Yang. Voucher specimens have been deposited in the Hainan branch of the Institute of Medicinal Plant Development, Chinese Academy of Medical Sciences (Herbarium No.: HNIMPLAD-DR-2023–001 to HNIMPLAD-DR-2023-087).

**Table 1:** Primer sequences and amplification programs for DNA barcode loci.

No.	Locus	Primer	Primer sequence (5’-3’)	Amplification programs	References
1	*trnP-psaJ*	trnP-psaJ-F	GTAGGGATGACAGGATTTGA	95°C 5min; [35cycles: 95°C 60s, 53°C30s, 72°C 80s]; 72°C 10min	Zhang et al., 2021
		trnP-psaJ-R	ACCATAGAGTAGTTAGCACA		
2	*psbK-psbI*	psbK-psbI-F	CGTAGATGTTATGCCTGTC	95°C 5min; [35cycles: 95°C 60s, 53°C30s, 72°C 80s]; 72°C 10min	Zhang et al., 2021
		psbK-psbI-R	AGAATCCGAAGATGAAGAGA		
3	*ITS2*	ITS2-F	GCGATACTTGGTGTGAAT	95°C 5min; [35cycles: 95°C 30s, 58°C30s, 72°C 40s]; 72°C 10min	Pang et al., 2020
		ITS2-R	GACGCTTCTCCAGACTACAAT		
4	*trnL-trnF*	trnL-trnF-F	CGAAATCGGTAGACGCTACG	95°C 5min; [35cycles: 95°C 60s, 58°C30s, 72°C 70s]; 72°C 10min	Pang et al., 2020
		trnL-trnF-R	ATTTGAACTGGTGACACGAG		
5	trnL(UAA)	trnL(UAA)-F	GGGCAATCCTGAGCCAA	95°C 5min; [35cycles: 95°C 60s, 55°C30s, 72°C 30s]; 72°C 10min	Wang et al., 2015
		trnL(UAA)-R	CCATTGAGTCTCTGCAC		

Additionally, relevant DNA barcode sequences were downloaded from the literature or NCBI (Samples 27–44 in **Supplementary Table S1**).

### 2.2 DNA extraction

Approximately 100 mg of fresh leaf tissue from each sample was used for DNA extraction with the Plant Genomic DNA Extraction Kit (OMEGA HP Plant DNA Kit, USA) according to the manufacturer’s protocol. Only DNA samples with A260/280 ratios between 1.8 and 2.0 and A260/230 ratios > 1.5 were used for downstream analyses.

### 2.3 PCR amplification and sequencing of DNA barcodes

The primers and PCR programs used for DNA barcode amplification are listed in **Table 1**. The PCR system (25 μL) consisted of 12.5 μL of 2×EasyTaq PCR SuperMix (TransGen Biotech, China), 1 μL each of the forward and reverse primers, and approximately 20 ng of DNA template. PCR products were detected via electrophoresis on 1% agarose gels. Successfully amplified products were purified and subjected to bidirectional sequencing by BGI Genomics Co., Ltd. Successful amplification was defined as the presence of a clear, single band of the expected size on 1% agarose gel. For sequencing, the concentration of PCR products was required to be ≥10–15 ng/μL, with a total amount of 50–100 ng per reaction. Bidirectional sequencing was performed. Mixed base calls (double peaks) were required to account for less than 20% of the total sequencing signal.

### 2.4 DNA barcode sequence analysis and phylogenetic tree construction

Successfully sequenced fragments were aligned using MEGA6.0. UPGMA (unweighted pair group method with arithmetic mean) phylogenetic trees were constructed based on the *trnP-psaJ and psbK-psbI* sequences.

### 2.5 Screening the SSR core primers

SSR sequences from the *Dracaena* transcriptome data previously obtained by our research group were analyzed and screened using MISA software. The screening criteria (Chu et al., 2025; Xu et al., 2024) for SSR loci were as follows: (1) removal of loci where the repeat unit consisted entirely of G/C bases; (2) removal of loci with mononucleotide or compound repeat units; (3) retention of SSR sequences with repeat numbers greater than 5; and (4) prioritized selection of loci from different gene sequences. Primers were designed from SSR loci meeting the aforementioned conditions under standard conditions. Subsequently, 192 primer pairs were randomly selected and synthesized using the adapter method, i.e., a 21 bp adapter sequence was added to the forward primer during synthesis. For PCR amplification using the adapter method, the first step involved the adapter-linked forward primer and the reverse primer binding to the template to produce a PCR product containing the adapter sequence. The second step involved a fluorescently labeled adapter primer and the reverse primer binding to the first-step PCR product to yield a fluorescently labeled PCR product with a 21 bp adapter sequence.

To estimate the genotyping error rate for SSR analysis, 10% of samples (n = 3) were selected for independent duplicate runs, prioritizing samples with complex stutter patterns, low peak heights, or ambiguous allele calls. Each duplicate run consisted of independent DNA re-extraction (where necessary), re-amplification by PCR, and re-analysis by capillary electrophoresis (CE). An error was defined as any discordance in allele size calls between the original and duplicate runs at any locus. The per-allele error rate was calculated as the number of discordant allele calls divided by the total number of allele comparisons (samples × loci × 2 alleles). The observed error rate was < 0.5% (e.g., 3 discordant calls out of 624 comparisons).

For alleles showing a size difference ≤ 2 bp that were initially ambiguous (e.g., overlapping bin boundaries or low signal-to-noise ratio), a second independent PCR and CE run was performed. If the duplicate run confirmed the same allele size (≤ 1 bp difference), the call was accepted. If the duplicate run yielded a discordant call (difference ≥ 2 bp), a third independent PCR was conducted. If still discordant after three runs, the genotype was recorded as missing data to avoid false allele assignments.

The 26 *Dracaena* samples were used as screening materials. PCR amplification was performed, followed by detection via agarose gel electrophoresis (1% gel), to select primers yielding clear, single, and nonsmearing bands. The PCR products generated using the selected primers were subsequently diluted to obtain fluorescent PCR products with uniform concentrations. These products were loaded onto a plate, denatured (95 °C, 3 min), and immediately cooled. According to the ABI 3730xL operational workflow, the SSR sample analysis program was run. The raw capillary electrophoresis data were analyzed using the fragment (plant) analysis function in GeneMarker software. By comparing the positions of the molecular weight internal standard with the peak positions of each sample in each lane, the fragment size of each amplicon was determined.

GenAlEx software version 6.501 was used to calculate genetic diversity indices for each SSR primer pair, including the number of observed alleles (Na), polymorphism information content (PIC), Shannon’s information index (I), observed heterozygosity (Ho), expected heterozygosity (He), and inbreeding coefficient (Fis). Furthermore, primers meeting the criteria of a good peak quality, an Na ≥ 4, and a PIC > 0.6 were selected to form a core SSR primer set with high polymorphism and resolution. MICRO-CHECKER software was used to test for null alleles and linkage disequilibrium. Loci showing potential null alleles.

### 2.6 Analysis of the genetic diversity of *Dracaena* germplasm resources based on SSR markers

Using DNA from all *Dracaena* samples extracted in Section 1.2 as a template, fluorescent PCR amplification was performed with the 16 core SSR primer pairs selected above. After amplification, 2 μL of the PCR product was separated by agarose gel electrophoresis (1% gel) and then diluted to obtain fluorescent PCR products with uniform concentrations. These products were denatured at 95 °C for 3 min, immediately cooled, and analyzed according to the ABI 3730xL operational workflow.

GenAlEx 6.5 was used to analyze the capillary electrophoresis data and determine amplicon fragment sizes. Based on the amplicon lengths for each SSR primer pair, programs such as PowerMarker 3.25, PopGene32, and NTsys2.1 were used to calculate genetic diversity indices and construct phylogenetic trees. AMOVA was performed using GenAlEx with 999 permutations to partition genetic variance among and within populations (**Supplementary Table 4**).

## 3 Results

### 3.1 Evaluating the DNA barcode fragments

Five previously reported candidate barcodes were evaluated in 26 *Dracaena* samples to identify suitable DNA barcode fragments for *Dracaena* authentication based on three aspects: the PCR amplification success rate, sequencing success rate, and number of variable sites (**Table 2**). The results showed that the *trnL-trnF* fragment had the lowest PCR amplification success rate (88%), while the trnL(UAA)-P6 fragment had the lowest sequencing success rate (26.7%); therefore, both were excluded. The PCR and sequencing efficiency of *ITS2* were also unsatisfactory. Through the variable site analysis, *trnP-psaJ and psbK-psbI were p*reliminarily selected as candidate DNA barcode sequences for the authentication of dragon’s blood source plants.

**Table 2:** Evaluation of the five DNA barcode loci.

DNA barcode loci	Number of individuals	Length/bp	Variable sites	PCR success/%	Sequencing success/%
*trnP-psaJ*	26	363	28	100	100
*psbK-psbI*	26	501	27	100	88
*trnL-trnF*	26	1059	30	88	82
*ITS2*	26	494	–	92	73
trnL(UAA)-P6	26	100	–	100	26.7

### 3.2 Identification using DNA barcodes

UPGMA phylogenetic trees based on *trnP-psaJ and psbK-psbI were c*onstructed using DNA barcode fragments obtained from the aforementioned 26 samples and 18 related sequences downloaded from the literature and NCBI database, respectively.

**Figure 1:**
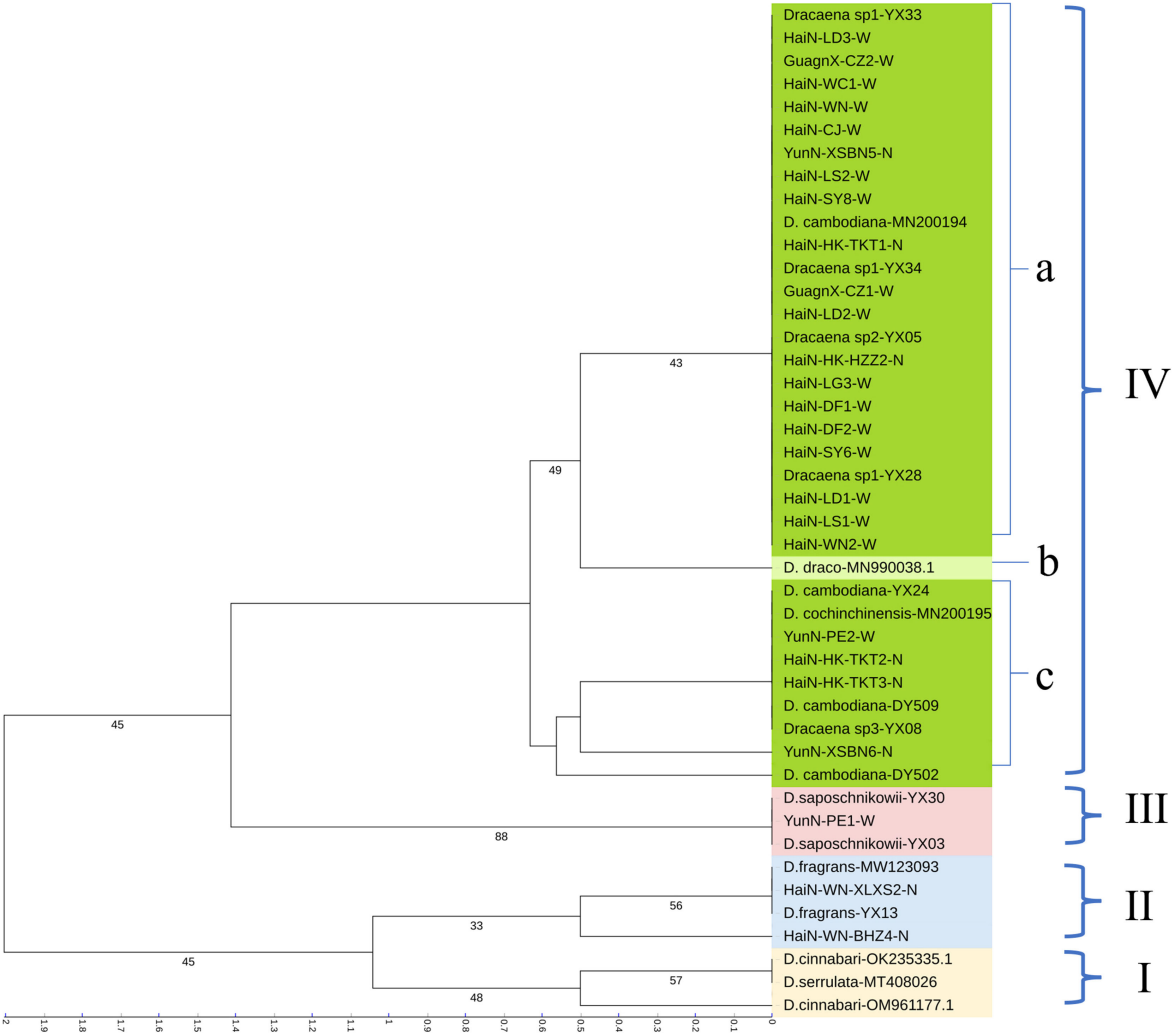
UPGMA trees constructed from the *trnP-psaJ barcode* loci.

In the *trnP-psaJ cluster* tree (**Figure 1**), at a genetic distance of 1.0, the *Dracaena* samples are primarily divided into four major branches. Branch I comprises *D. cinnabari* and *D. serrulata* from the Arabian Peninsula. Branch II contains *D. fragrans* (XLS) and D. reflexa (BHZ) samples, where the *D. fragrans* (XLS) samples cluster with previously reported sequences (*D. fragrans*-MW123093.1 and YX13), verifying the accuracy of species identification. The sample from Yunnan (PE1) in this study and the previously reported D. saposchnikowii-YX30/YX03 (“*Yanzong*”) cluster on Branch III. Branch IV is further subdivided into three subbranches at a distance of 0.5. All the wild *D. cambodiana* samples cluster on subbranch a. The samples from Laos (*Dracaena* sp2-YX05), Guangxi (GuangX-CZ1-W, GuangX-CZ2-W), and Yunnan (XSBN-5), as well as the horticultural varieties “Heizhenzhu” (HaiN-HK-HZZ2) and “Taikongtie” (HaiN-HK-TKT1) are also found on this subbranch. *D.draco* is separately located on subbranch b. Subbranch c contains two Yunnan *Dracaena* samples (YunN-XSBN6 and PE2), the horticultural variety “Taikongtie” (HaiN-HK-TKT2 and HaiN-HK-TKT3), and previously reported sequences such as *D. cambodiana*-YX24, DY509, and *D. cochinchinensis*-MN200195.

**Figure 2:**
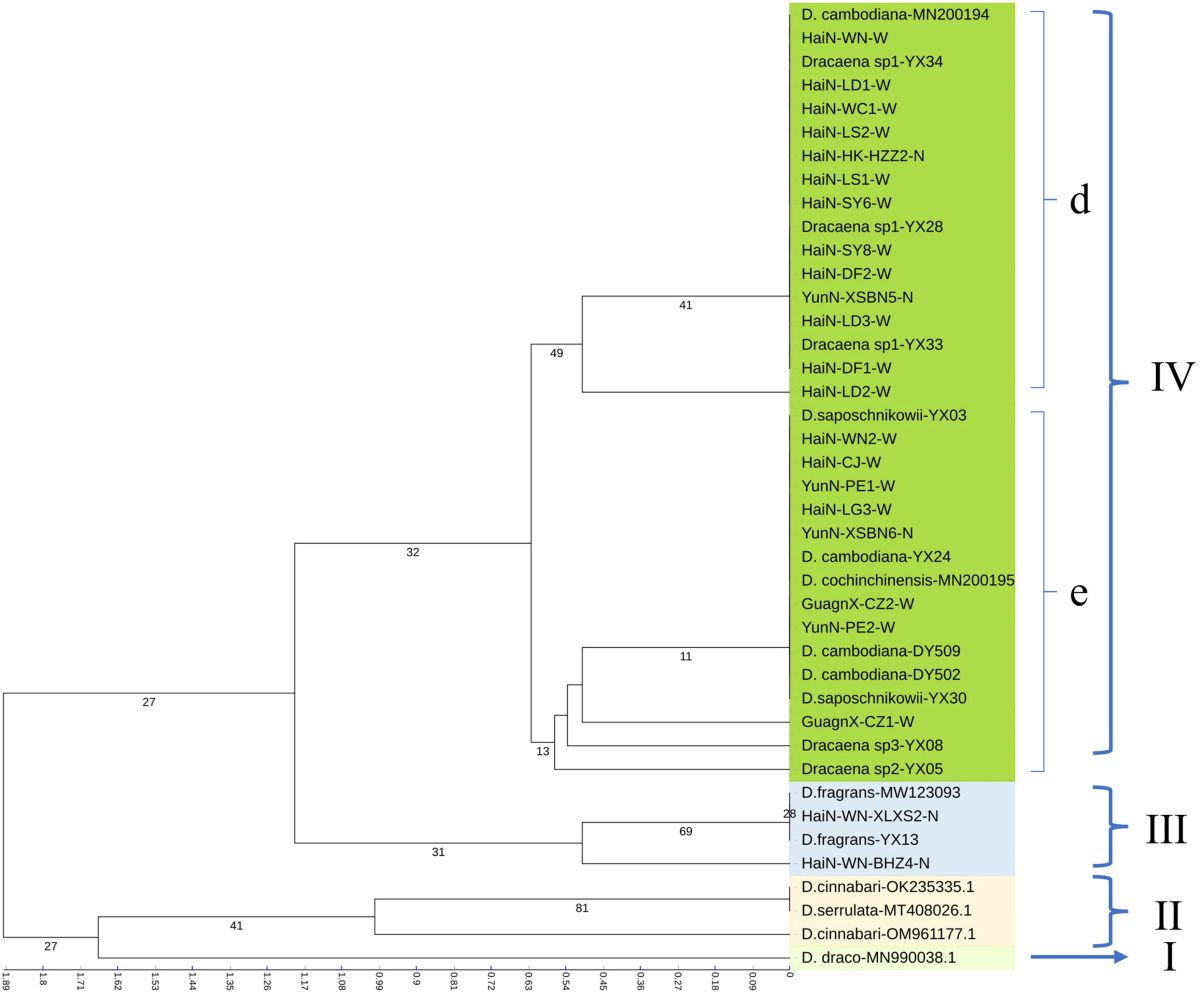
UPGMA trees constructed from the *psbK-psbI* barcode loci.

The clustering results for *psbK-psbI (***Figure 2**) were similar to those for trnP-psaJ. The domestic *Dracaena* group (**Figure 2**, IV) was distinguished from foreign groups (**Figure 2**, I, II) and the outgroup (**Figure 2**, III). However, two notable inconsistencies were observed in the identification of domestic *Dracaena* samples: (1) The 14 wild *D. cambodiana* samples clustered together in the *trnP-psaJ tree (***Figure 1A**), but in the *psbK-psbI tree*, these samples split into two branches. Three wild *D. cambodiana* samples (HaiN-CJ-W, HaiN-LG3-W, and HaiN-WN2-W) clustered with *D. cochinchinensis*-MN200195 (**Figure 2E**), while the other 11 samples clustered on another branch (**Figure 2D**). (2) D. saposchnikowii-YX30/YX03 (“*Yanzong*”) formed a relatively independent cluster in the *trnP-psaJ tree (***Figure 1**, III), but in the *psbK-psbI tree*, this cluster mixed with some wild *Dracaena* samples from Hainan and Guangxi (GuangX-CZ1-W, GuangX-CZ2-W) (**Figure 2E**). Additionally, *psbK-psbI sequen*ces for the three “Taikongtie” (TKT) horticultural varieties could not be assembled because of the poor sequencing chromatogram quality.

Barcoding gap analysis was conducted for both the trnP-psaJ and psbK-psbI regions using iTaxoTools. The analysis reveals contrasting patterns of genetic divergence. Comparisons between the outgroup species (Dracaena fragrans and Dracaena reflexa) and the Dracaena species examined show substantial genetic distances (e.g., K2P distances up to 0.0117 for trnP-psaJ), indicating clear differentiation between these groups. However, within the Dracaena species complex that produces Dragon’s Blood — including D. cambodiana, D. cochinchinensis, and “Yanzong” — interspecific distances are considerably low and overlap extensively with intraspecific distances, failing to satisfy the strict criterion for a barcoding gap. This low level of genetic divergence is biologically meaningful: it is consistent with the hypothesis that these species have undergone recent radiation and maintain chemical similarity in their red resin, which is the basis for their traditional use as interchangeable sources of Dragon’s Blood. Thus, while the selected barcodes can effectively distinguish Dracaena from outgroups, they lack sufficient resolution for species-level discrimination within the Dragon’s Blood source complex. The detailed pairwise genetic distance matrix is provided in **Supplementary Table 3** (see sheets trnP-psaJ and psbK-psbI).Therefore, this study further employed SSR molecular marker technology to focus on the *Dracaena* species distributed within China.

### 3.3 Screening SSR core primers for *Dracaena*

**Figure 3:**
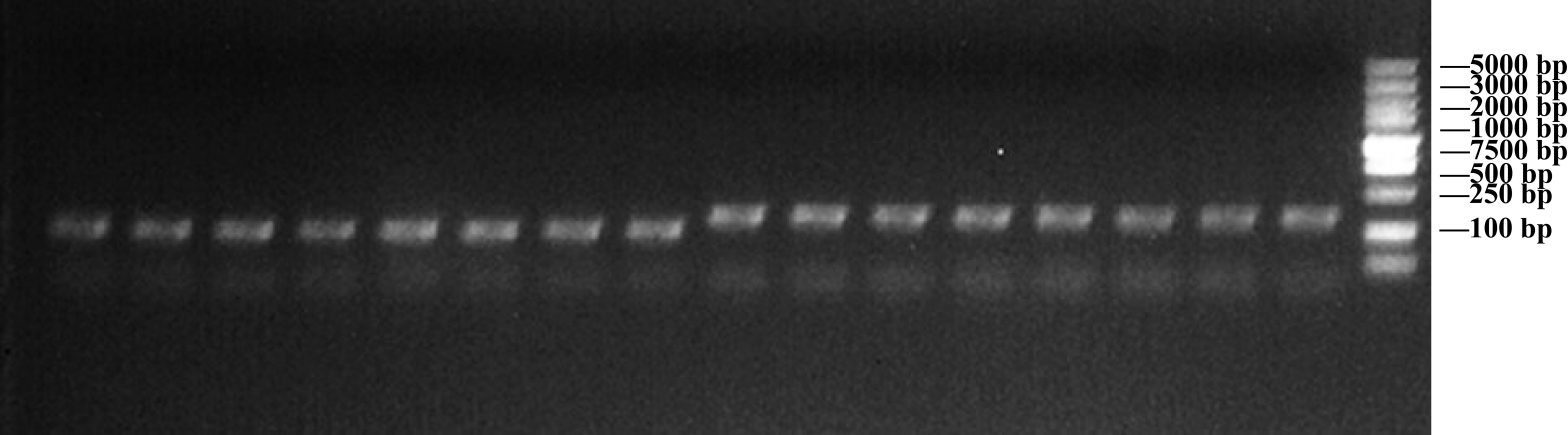
Image of a gel containing some of the amplification products from 2 SSR loci in 8 samples.

Using bioinformatics methods, 192 SSR primer pairs meeting the preliminary criteria were selected from the *Dracaena* transcriptome. These 192 primers were further experimentally screened using the 26 samples mentioned above. Based on criteria such as clear, single, nonsmearing amplicon bands (**Figure 3**), a number of alleles (Na) ≥ 4, a polymorphism information content (PIC) > 0.6, and an amplicon length between 110 ~ 350 bp, 16 pairs of SSR core primers were ultimately selected (**Table 3**). As shown in **Table 3**, the SSR primers amplified an average of 6.13 loci. The PIC values ranged from 0.607 to 0.796. The average gene diversity index (Hs) was 0.8533, and the average Shannon diversity index (I) was 1.4683. The expected heterozygosity (He) for each primer was greater than the observed heterozygosity (Ho). These data indicate that the 16 SSR primers possess good specificity and high polymorphism, making them suitable as SSR molecular marker primer sets for analyzing genetic relationships in *Dracaena*. Amplification success rates (transferability) ranged from 96.55% to 100% across all samples, including outgroup species (**Supplementary Table 2**).

**Table 3:** SSR molecular marker primer sets and their characteristics in *Dracaena* species.

No.	Primer name	SSR repeat	Primer sequence (5’-3’)	Size/bp	Na	PIC	I	Ho	He	Hs
1	DSSR-080	(CGCCT) 5	F:TCTTCGTGGAGGCCCTGATAR:GGGGAAGGCAAAGGAGAGA	268	8	0.796	1.85	0.615	0.82	0.91
2	DSSR-050	(GCTA) 6	F:CCGACCATGGACAAGAAGCT R:AGTTTGCAACCTGGGTTCCA	277	7	0.789	1.797	0.077	0.813	0.907
3	DSSR-063	(AAAAG) 7	F:TCTTGATCTTCCGGCAGCAG R:TGCACCTTGGCTTCGTAGAG	252	5	0.734	1.53	0.538	0.772	0.886
4	DSSR-174	(ACG) 6	F:AGGCCCACACTTTTCCCAAA R:GCCAAACAATAACGAGCCCC	223	5	0.718	1.509	0.385	0.754	0.877
5	DSSR-087	(TTAT) 5	F:TGGTGAAGATGTTGGCCTCC R:AGGGGCAGCACATTTCTCTC	253	6	0.686	1.451	0.692	0.734	0.867
6	DSSR-187	(ACT) 6	F:CGCCATGCTCAAGTCCTACT R:GACCCACAGCTCATCCATCC	268	7	0.674	1.514	0.385	0.71	0.855
7	DSSR-086	(TTTA) 5	F:CATGCGGCCATTATTGTGGG R:CCACTGCTCTTCCAAGGGAA	269	8	0.666	1.544	0.308	0.695	0.848
8	DSSR-056	(TCTA) 5	F:GCTTAAAGGGTGTGCTTGGC R:GGCTTGCAGGAATTTCTGGC	234	5	0.656	1.363	0.308	0.698	0.849
9	DSSR-039	(AGCC) 7	F:TTGACGTTCATGTCCCTGCA R:AGAAACAGTTCAGCTCGGCA	246	7	0.649	1.496	0.077	0.672	0.836
10	DSSR-013	(AAAAG) 5	F:TGCCCTGTTGTTTTCTTCGT R:GTTTGTAGTGCCCACCCTGT	261	8	0.644	1.514	0.154	0.669	0.835
11	DSSR-079	(AGAA) 5	F:AAAACCATAGCCACGACCGT R:GCAGGCCCTGTAGTTGGATT	172	6	0.63	1.389	0.077	0.66	0.83
12	DSSR-065	(TTAT) 5	F:TCCCTAATGGTACCTCGCCA R:GGAGGTTTTTACCGGCAGGA	220	5	0.629	1.329	0.231	0.672	0.836
13	DSSR-006	(AACCA) 5	F:ATAATCCCCGGAGCTAACGC R:TGATTAGGGCGACGGATTGG	160	6	0.62	1.356	0.154	0.654	0.827
14	DSSR-026	(TTTTC) 5	F:ACAAGTGTGAGCCTTGGGAC R:TCACGACGATTGATCCACAA	270	6	0.62	1.356	0.154	0.654	0.827
15	DSSR-022	(ATGA) 5	F:CCACTGTTGGTCATGCTTCT R:CAACCTGTTGAGGCAGAGGT	220	5	0.615	1.305	0	0.651	0.826
16	DSSR-188	(TCT) 5	F:TAGAACGGCACTGAACCCAG R:TTCAACAGAACTGCCTGCCA	243	4	0.607	1.189	0.308	0.671	0.836

Na, Number of alleles; PIC, Polymorphism information content; I, Shannon’s information index; Ho, Observed heterozygosity; He, Expected heterozygosity; Hs, Expected heterozygosity within subpopulations.

### 3.4 Clustering analysis of the 26 *Dracaena* samples based on the core SSR markers

Pairwise linkage disequilibrium (LD) tests were performed for all 16 SSR loci using PowerMarker software. After applying Bonferroni correction for multiple comparisons (α = 0.05/120 = 0.000417), most locus pairs exhibited significant LD (p < 0.000417). Only three locus pairs showed no significant LD after correction: LXS026–LXS039 (p = 0.00138), LXS039–LXS050 (p = 0.00168), and LXS087–LXS174 (p = 0.8657). Detailed results are provided in **Supplementary Table 6**.

Due to the presence of significant LD among most locus pairs, we did not use all 16 loci directly for population structure analysis. Instead, we applied a data filtering strategy to reduce redundancy: for strongly linked locus pairs (e.g., LXS013–LXS022, LXS079–LXS080, LXS187–LXS188), we retained only one locus per pair. The final subset of 13 relatively independent loci (LXS006, LXS013, LXS026, LXS039, LXS050, LXS056, LXS063, LXS065, LXS079, LXS086, LXS087, LXS174, LXS187) was used for subsequent analyses.

**Figure 4:**
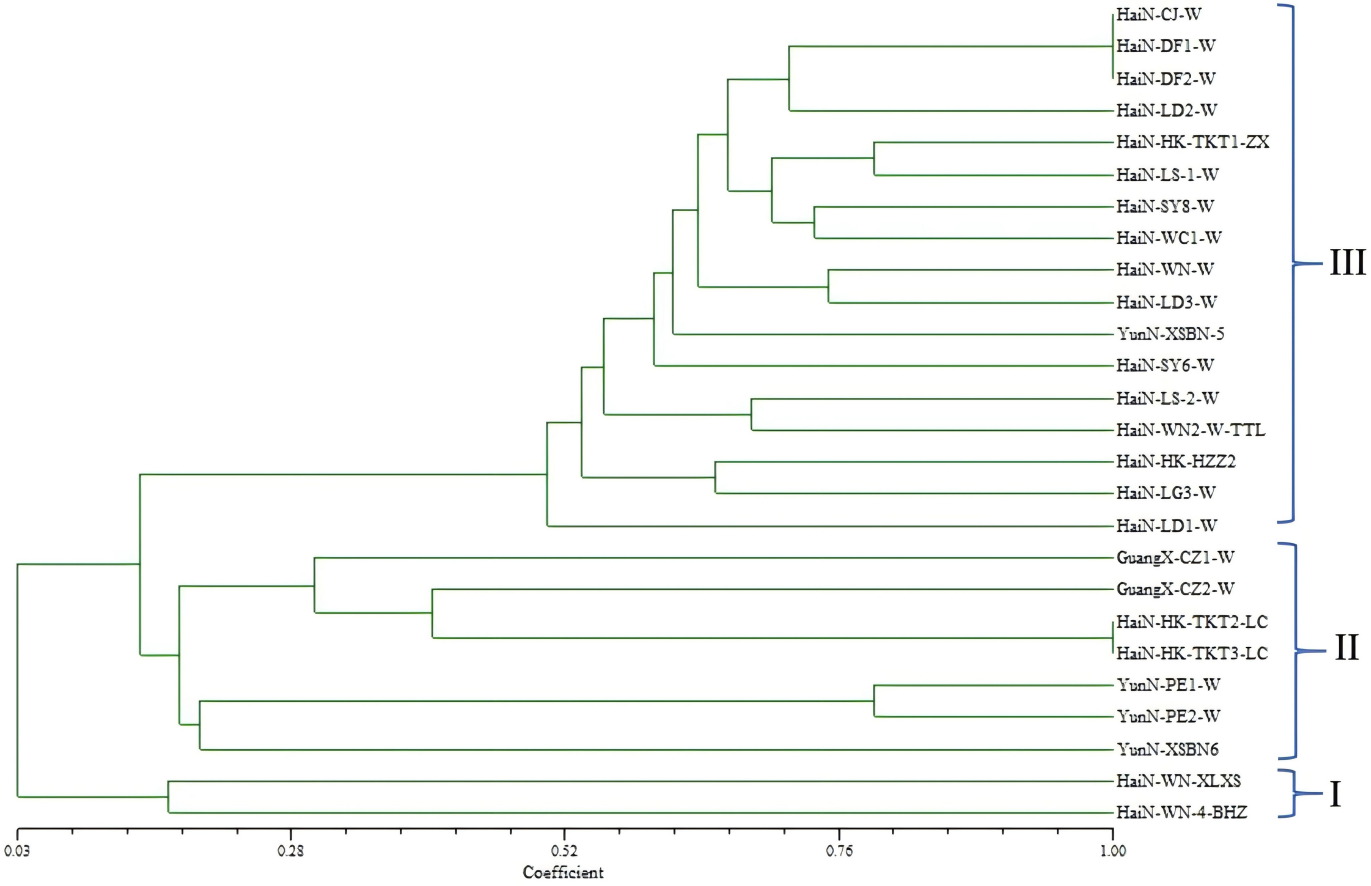
Cluster dendrogram of 26 *Dracaena* samples constructed using the 13 core SSR primers.

A clustering analysis was performed on the aforementioned 26 samples using the final set of 13 SSR markers selected after LD-based filtering. The cluster tree (**Figure 4**) shows that D. reflexa (BHZ) and *D. fragrans* (XLXS) form a distinct branch (**Figure 4**, I) that is clearly separated from the domestic dragon’s blood source plants. The domestic source plants are further divided into two branches. All *D. cambodiana* samples cluster on one branch (**Figure 4**, III). The horticultural variety “Taikongtie” (TKT2 and TKT3) first clusters with two *Dracaena* samples from Guangxi (GuangX-CZ1-W, GuangX-CZ2-W) and then with samples from Yunnan (XSBN-5, PE1 and PE2) on the other branch (**Figure 4**, II).

### 3.5 Identification and analysis of cultivated *Dracaena* in China

Currently, *Dracaena* trees are widely cultivated as landscaping or indoor ornamental plants in China. Samples were collected from Hainan, Guangdong, Yunnan, Hunan, Shandong, and other locations to clarify the species of these cultivated plants. They were identified using the *trnP-psaJ fragmen*t and the 13 core SSR primers described above.

**Figure 5:**
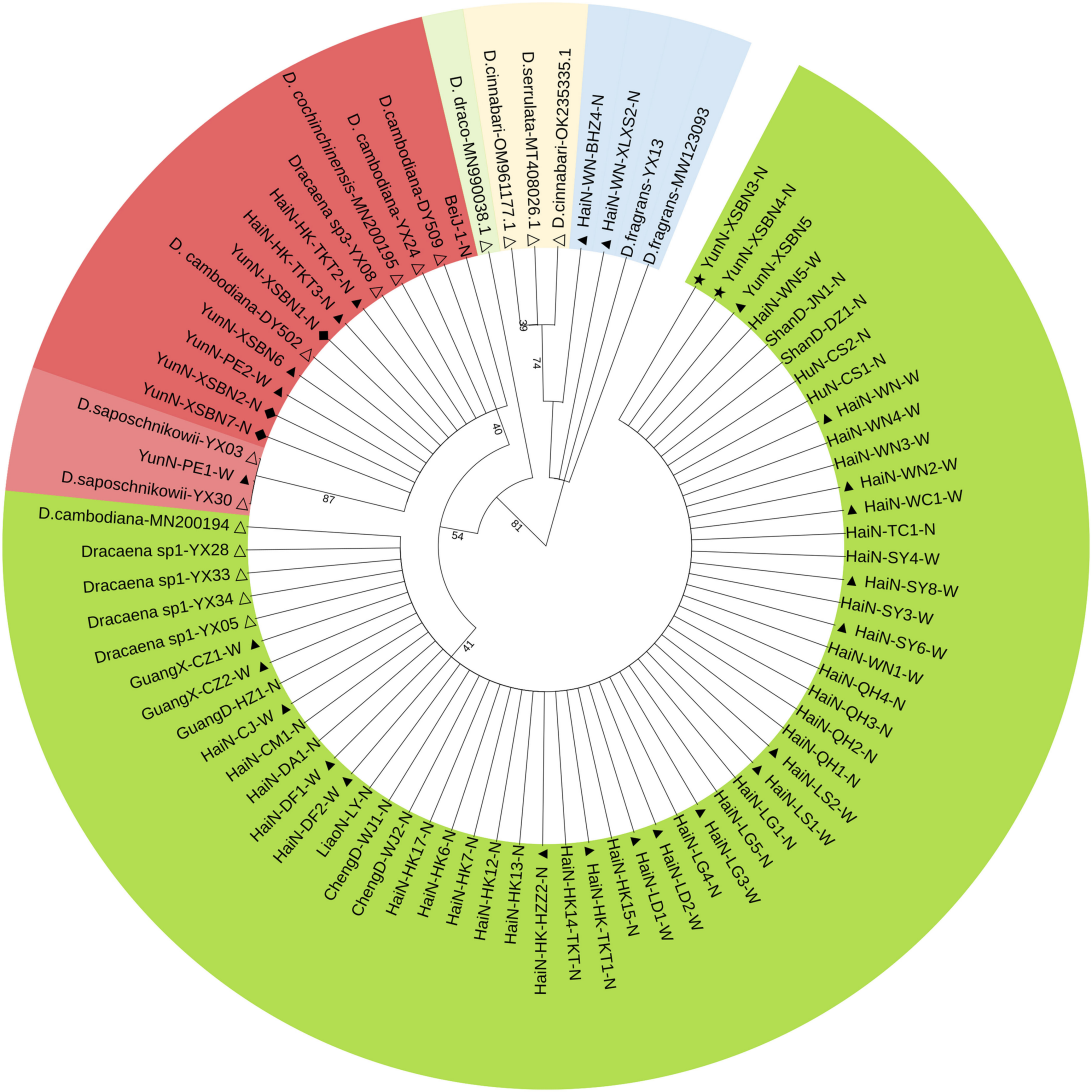
Barcode identification of cultivated *Dracaena* plants using the *trnP-psaJ region.* (Triangles represent samples from **Figure 1**; solid triangles denote samples from this study, while open triangles represent downloaded sequences.).

In the *trnP-psaJ fragmen*t cluster tree (**Figure 5**), landscaping *Dracaena* trees from Hainan, Guangdong, Hunan, Shandong, Liaoning, etc., cluster with wild *D. cambodiana* (green section in **Figure 5**). Yunnan samples can be divided into two groups: one group (YunN-XSBN3,4) (pentagon symbols in **Figure 5**) clusters with wild *D. cambodiana* (green section), while another group (YunN-XSBN1,2 and 7) (diamond symbols in **Figure 5**) clusters with the horticultural variety “Taikongtie” (TKT2 and TKT3) (dark red section) and then with the “*D.draco*” plant (pink section).

**Figure 6:**
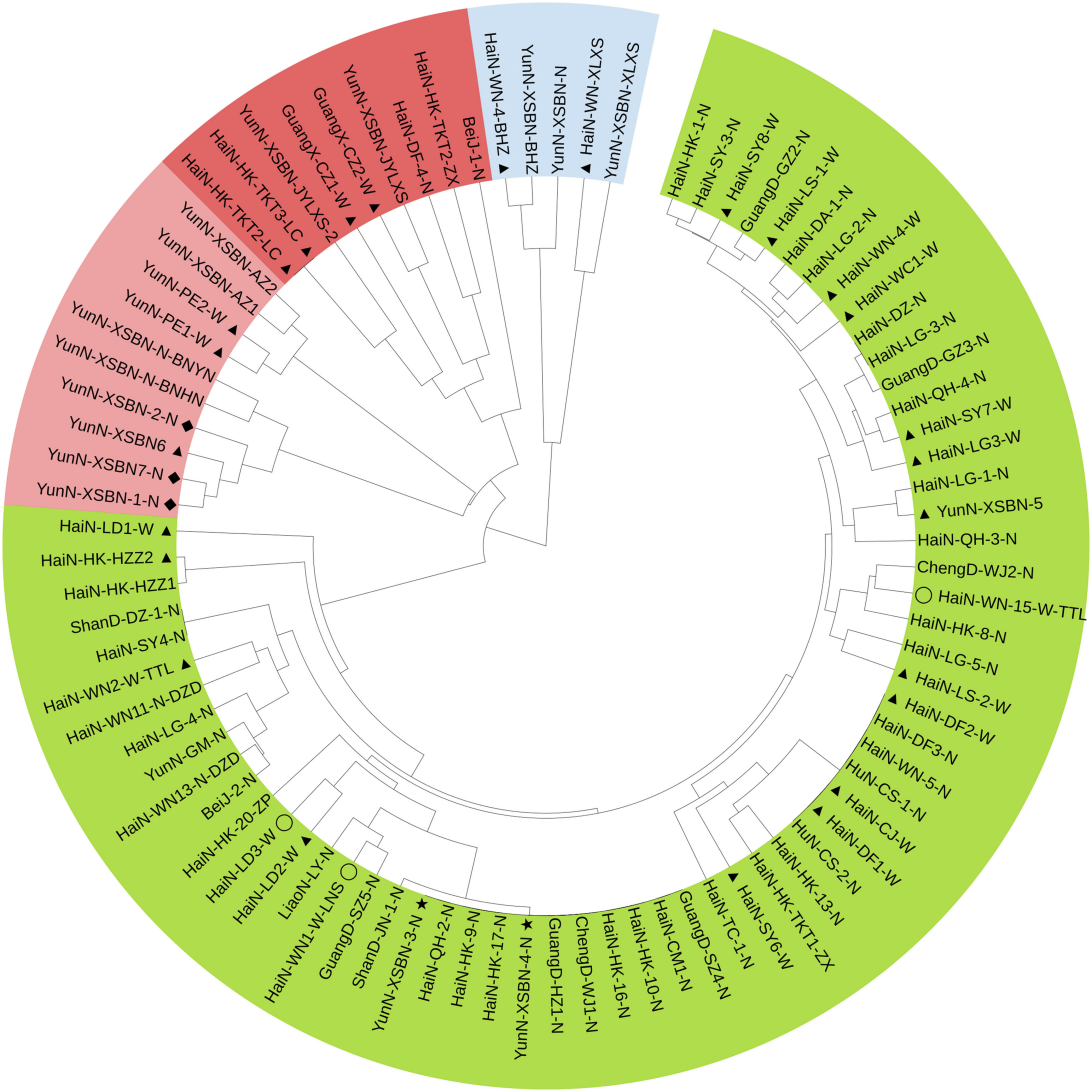
SSR identification of cultivated *Dracaena* species.

In the SSR cluster tree (**Figure 6**), the clustering pattern of these cultivated *Dracaena* trees is largely consistent with the DNA barcode results. Landscaping trees from Hainan, Guangdong, Hunan, Shandong, Liaoning, etc., cluster with wild *D. cambodiana* in a large branch (green section in **Figure 6**). Yunnan samples are similarly divided into two groups: one clusters with *D. cambodiana* (green section, pentagon), while more Yunnan samples cluster with the “*D.draco*” plant (red section, diamond). Notably, the two samples from Guangxi (GuangX-CZ1-W and GuangX-CZ2-W) cluster with wild *D. cambodiana* in the *trnP-psaJ* tree *(g*reen in **Figure 5**), but in the SSR tree, these two samples cluster with the horticultural variety “Taikongtie” (dark red in **Figure 6**).

**Figure 7:**
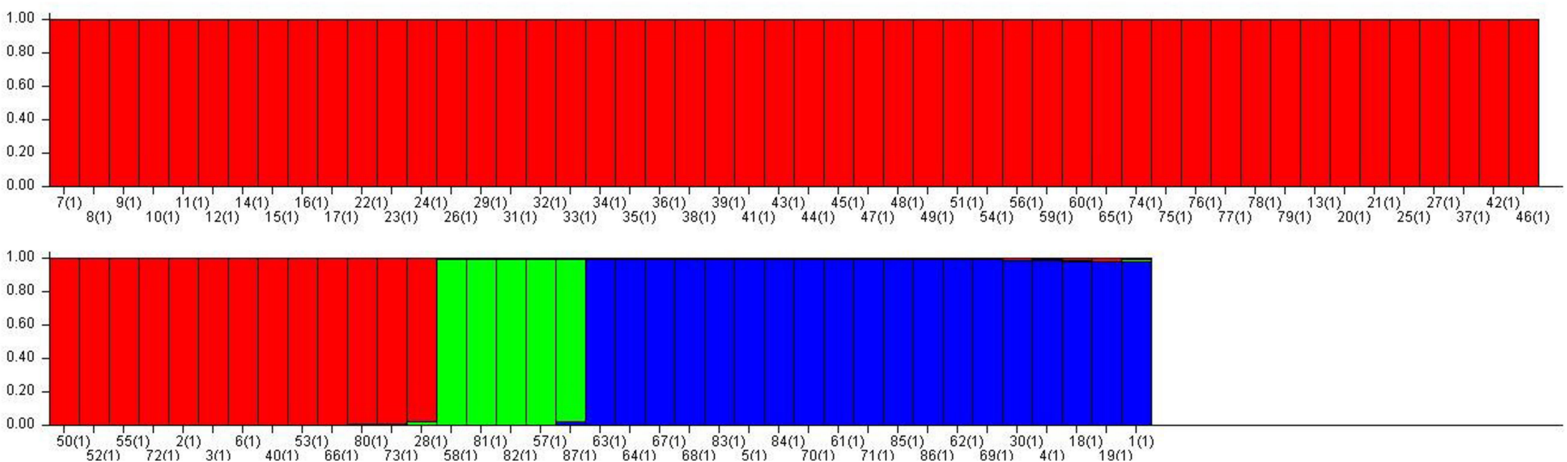
STRUCTURE analysis of 87 *Dracaena* samples at K = 3.

STRUCTURE analysis was performed to further investigate the genetic structure and possible admixture among the 87 samples(**Figure 7**). At K = 3, the clustering results are as follows: the outgroups *Dracaena reflexa* (samples 58 and 82) and *Dracaena fragrans* (samples 57, 81, and 87) formed one cluster (green); “Taikongtie” (samples 18, 19, and 30), “*Yanzong*” (samples 69 and 70), and *Dracaena cambodiana* from Yunnan formed a second cluster (blue); all remaining samples grouped with *Dracaena cambodiana* from Hainan into a third cluster (red). Notably, “*Taikongtie*” did not show evidence of mixed ancestry; instead, it clustered together with “*Yanzong*” and Yunnan *D. cambodiana*.

## 4 Discussion

### 4.1 Combined DNA barcoding and SSR markers: an efficient and accurate strategy for identifying closely related species

DNA barcoding has become a vital tool for species identification due to its standardization and reliance on reference databases. However, unlike in animals, no single “universal” DNA barcode fragment is ideal for all plant groups (CBOL Plant Working Group, 2009). The choice of fragment(s) strongly depends on the specific plant group and identification purpose (Liu et al., 2025). In this context, five DNA barcode fragments previously reported as suitable for *Dracaena* identification were evaluated in this study (Wang et al., 2015; Takawira-nyenya et al., 2018; Zhang et al., 2021; Pang et al., 2020). Based on the amplification and sequencing success rates, trnL-trnF, ITS2, and trnL(UAA)-P6 were excluded. Combined with sequences from public databases, our results indicate that *trnP-psaJ and psbK-psbI can cl*early distinguish foreign species (*D. cinnabari* and *D.draco*) from domestic dragon’s blood source plants. However, minor inconsistencies were observed when distinguishing between *D. cochinchinensis*, *D. cambodiana*, and the “*D.draco*” plant. In contrast, SSR markers, with their characteristics of multiple alleles, codominance, and high polymorphism, show significant advantages in revealing the intraspecific genetic structure and discriminating closely related species or even cultivars. Although SSR markers for *Dracaena* have been reported previously, they have focused mostly on characterization, with no reports of their use for systematic germplasm resource identification. This study is the first to screen and validate 16 SSR primers suitable for dragon’s blood source plants. The clustering analysis using these primers clearly distinguished the closely related *D. cambodiana*, *D. cochinchinensis*, and the “*D.draco*” plant with the results corroborating the overall classification framework of *trnP-psaJ* but offering higher resolution.

In practice, DNA barcoding and SSR technologies each have advantages for authenticating dragon’s blood source plants. DNA barcodes allow comparisons with reference sequences from NCBI or the literature to verify clustering and identification results, whereas SSR markers excel at discriminating closely related species and even intraspecific variants. Therefore, we propose an integrated strategy of “preliminary screening and positioning using the *trnP-psaJ barcode*, followed by fine-scale discrimination using SSR markers”. Their combination offers both efficiency and precision, providing a reference molecular authentication protocol not only for dragon’s blood source plants but also for other taxonomically challenging groups of closely related species.

### 4.2 Re-evaluating taxonomic controversies surrounding dragon’s blood source plants

In this study, we observed discordant clustering patterns between DNA barcodes (chloroplast *trnP-ps*aJ and p*sbK-psb*I) and SSR markers (nuclear) for certain *Dracaen*a taxa, particularly involving *D. cochinchinensis*, the “*Yanzong*” plant, and *Guangxi* populations (**Figures 1**, **2** vs. **Figure 4**). Specifically, while chloroplast barcodes showed inconsistent or unresolved clustering among these groups, SSR analysis clearly segregated them into distinct genetic clusters corresponding to geographic origins (*Yunnan* vs. *Guangxi*).

We acknowledge that this observation remains descriptive rather than statistically confirmed. Several limitations constrain our ability to interpret this discordance. First, our sample size is modest (n = 26), and some taxonomic subgroups are represented by only two individuals (e.g., *D. cochinchinensis*, Guangxi populations), which increases the risk of sampling artifacts. Second, geographic sampling is uneven: Hainan populations are well-represented, while those from Yunnan, Guangxi, and Laos are sparsely sampled, potentially inflating or obscuring true discordance patterns. Third, the two marker systems differ fundamentally in data type (chloroplast sequences vs. nuclear SSR fragment lengths), making direct quantitative comparison challenging.

Potential biological explanations for such discordance—if real—include incomplete lineage sorting (if divergence among these taxa was rapid), hybridization or introgression (which would affect nuclear and chloroplast genomes differently), and different modes of inheritance (maternal for chloroplasts vs. biparental for nuclear SSRs). However, given the limitations noted above, we refrain from favoring any single mechanistic interpretation.

### 4.3 Implications for the resource status, conservation, and development

The stark reality of wild *Dracaena* conservation underpins the urgency of our work. All major source species are threatened: *D. cinnabari* and *D.draco* are internationally listed as vulnerable or endangered by the IUCN, while *D. cochinchinensis* and *D. cambodiana* are nationally protected in China (Zhang et al., 2021; Xin et al., 2024). This critical status, compounded by the genus’s slow growth and prolonged resin production cycle, makes the development of cultivated alternatives a conservation and economic imperative.

Our molecular identification strategy directly supports this goal. By reliably distinguishing species and varieties, it enables the accurate characterization and management of exsitu germplasm collections. Crucially, the finding that *D. cambodiana* forms the backbone of Chinese cultivation provides a clear target for breeding and domestication programs. Future efforts should prioritize the systematic evaluation of resin yield and quality across identified germplasms—including *D. cambodiana* and varieties like “Heizhenzhu” and “Taikongtie”—to select superior genotypes for sustainable plantation forestry. Thus, this study bridges taxonomic clarification with practical application, offering a molecular toolkit to guide the conservation, breeding, and sustainable commercial development of dragon’s blood resources.

We acknowledge that the present study does not directly authenticate commercial resin samples. While we attempted DNA extraction from resin, repeated failures due to DNA degradation and PCR inhibition prevent us from validating our markers on processed products. Future studies should explore chemical fingerprinting (e.g., HPLC or LC-MS) as a complementary approach for resin authentication. Additionally, the taxonomic conclusions regarding Hainan populations require validation with broader geographic sampling and phylogenomic data.

## 5 Conclusion

This study resolves long-standing taxonomic ambiguities surrounding dragon’s blood source plants by establishing and validating a novel, two-tiered molecular authentication system. The system integrates the optimized chloroplast barcode *trnP-psaJ for rap*id taxonomic positioning with a panel of 16 SSR markers for high-resolution genotyping. This combined approach successfully discriminated all major sources—*D.cinnabari*, *D.draco*, *D. cochinchinensis*, *D. cambodiana*, and the “*D.draco*” plant—and provided robust evidence supporting the genetic affinity of Hainan populations with *D. cambodiana*, although formal taxonomic revision requires additional evidence. Furthermore, it revealed that *D. cambodiana* constitutes the primary cultivated germplasm in China, clarifying the genetic identity of key horticultural varieties.

By delivering precise molecular tools for species identification and germplasm characterization, this work provides a critical foundation for ensuring the traceability and quality control of dragon’s blood, guiding the conservation of wild populations, and facilitating the strategic breeding of high-yielding cultivars for sustainable utilization.

## Data Availability

The datasets presented in this study can be found in online repositories. The names of the repository/repositories and accession number(s) can be found in the article/**Supplementary Material**.
